# Percutaneous transhepatic cholangiobiopsy

**DOI:** 10.1590/0100-3984.2017.0228

**Published:** 2019

**Authors:** Thiago Franchi Nunes, Tiago Kojun Tibana, Rômulo Florêncio Tristão Santos, Bernardo Bacelar de Faria, Edson Marchiori

**Affiliations:** 1 Universidade Federal de Mato Grosso do Sul (UFMS), Campo Grande, MS, Brazil.; 2 Santa Casa de Campo Grande, Campo Grande, MS, Brazil.; 3 Universidade Federal do Rio de Janeiro (UFRJ), Rio de Janeiro, RJ, Brazil.

**Keywords:** Bile duct neoplasms, Biliary tract, Biopsy/methods, Biopsy, needle/methods, Cholangiography, Neoplasias dos ductos biliares, Sistema biliar, Biópsia/métodos, Biópsia por agulha/métodos, Colangiografia

## Abstract

Most tumors of the biliary tract are too small to have specific imaging characteristics or for percutaneous puncture to provide sufficient material for diagnosis. Percutaneous transhepatic biliary drainage, in addition to being a well-established technique in the treatment of obstructive jaundice, provides adequate access for sampling obstructive lesions. In cases of biliary lesions, percutaneous transhepatic biopsy of the biliary tract has proven to be a useful diagnostic technique, with a reported accuracy of over 90% at some referral centers.

## Abstract (pt)

Os tumores das vias biliares são, em sua maioria, muito pequenos para apresentarem características específicas por imagem ou para permitir punção percutânea com material suficiente para o diagnóstico. A drenagem biliar trans-hepática percutânea, além de ser uma técnica bem estabelecida no tratamento de icterícia obstrutiva, fornece um acesso adequado para amostragem de lesões obstrutivas. Nos casos de lesões biliares, a colangiobiópsia transbiliar percutânea demonstra ser uma boa técnica diagnóstica, com acurácia ultrapassando 90% em alguns centros de referência.

## INTRODUCTION

Bile duct tumors are, for the most part, too small to have specific imaging characteristics or to allow percutaneous puncture that collects sufficient material for diagnosis^(^1^,^^2^^)^. In addition, malignant neoplasms are not easily distinguished from benign lesions on the basis of the pathology findings. The results of fine-needle aspiration biopsy (FNAB) of the biliary system are inferior to those of FNAB of other sites^(^2^)^. Therefore, other techniques for obtaining histological samples have been developed for use in the biliary tract^(^3^-^10^)^.

Percutaneous transhepatic biliary drainage (PTBD), in addition to being a well-established technique for the treatment of obstructive jaundice, provides adequate access for the sampling of obstructive lesions. In cases of biliary lesions, forceps biopsy during PTBD is a good diagnostic technique, with a reported accuracy of over 90% at some referral centers^(^3^,^^4^^)^.

Prior to any approaches to the biliary tract, it is paramount to have in-depth knowledge in the interpretation of imaging tests, especially magnetic resonance cholangiography^(^2^,^^11^^,^^12^^)^. Possible biliary or vascular anatomical variants should be identified, the degree of biliary obstruction should be determined, and the extent of tumor invasion toward the hepatic hilum, according to the Bismuth classification^(^13^)^. Patients who benefit most from forceps biopsy during PTBD are those with a serum bilirubin level > 10 mg/dL and an obstruction of the upper biliary tract, in the hepatic hilum (Klatskin-like lesion), that is classified as Bismuth type II, III, or IV, involves more than two thirds of the circumference of the bile duct, and has long (> 2 cm) stenoses.

## PROCEDURE

**Figure 1:**
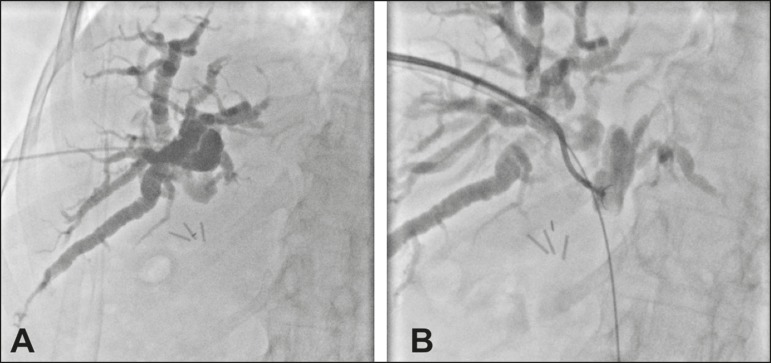
**A:** Puncture of the right bile duct in a patient with Bismuth type II stenosis. **B:** Passage of a 0.035-in guidewire, placement of a 9F sheath, and throughthe- needle forceps biopsy. The pathology report revealed cholangiocarcinoma.

**Figure 2:**
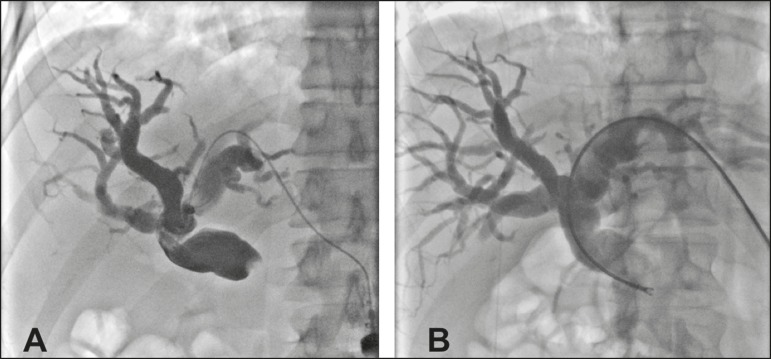
**A:** Puncture of the left bile duct in a patient with Bismuth type I stenosis. **B:** Placement of an 8F sheath and through-the-needle forceps biopsy with no guidewire. The pathology report revealed liver metastasis of colorectal carcinoma.

Initially, puncture of the bile duct (right or left), based on previous imaging examinations, is performed. That is followed by cholangiography with a right anterior oblique projection, the objective of which is to visualize the point of obstruction, as well as the morphology and extent of the stenosis. If possible, fiberoptic endoscopy is used in order to characterize the target lesion more accurately. After passing through the stenosis, an angled introducer sheath is implanted in the region to be biopsied. If a 9F sheath is chosen, the corresponding guidewire should be 0.035 in. long, whereas it should be 0.014 in. long if an 8F sheath is chosen. With endoscopic forceps, at least five fragments of various sizes are collected from the perihilar region lesion (Figures 1 and 2). At the end of the procedure, it is recommended that a biliary drain (with its distal end in the duodenum) be inserted or that external drainage be performed, in case there are technical difficulties in bypassing the stenosis.

Forceps biopsy during PTBD is a technically simple, minimally invasive procedure, with low complication rates and high diagnostic success rates in comparison with other known techniques^(^1^,^^4^^)^. Its use has expanded the scope of research into biliary diseases. In clinical practice, it has proven to be an accurate, reliable method for the histopathological diagnosis of biliary tumors, as well as having a wide range of other applications.
